# Spectral Domain-Optical Coherence Tomography As a New Diagnostic Marker for Idiopathic Normal Pressure Hydrocephalus

**DOI:** 10.3389/fneur.2017.00172

**Published:** 2017-05-01

**Authors:** Joana M. Afonso, Manuel Falcão, Frank Schlichtenbrede, Fernando Falcão-Reis, Sérgio Estrela Silva, Till M. Schneider

**Affiliations:** ^1^Department of Ophthalmology, São João University Hospital, Porto, Portugal; ^2^Department of Ophthalmology, University Hospital Mannheim, Mannheim, Germany; ^3^Faculty of Medicine, Department of Sense Organs, University of Porto, Porto, Portugal; ^4^Department of Neuroradiology, University Hospital Heidelberg, Heidelberg, Germany

**Keywords:** idiopathic normal pressure hydrocephalus, spectral domain-optical coherence tomography, enhanced depth imaging, choroidal thickness, retinal nerve fiber layer

## Abstract

**Purpose:**

Characterized by a progressive onset of gait disturbances, dementia, and urinary incontinence, idiopathic normal pressure hydrocephalus (iNPH) is considered a rare, but under-diagnosed disease. Non-invasive diagnostic markers are still insufficient to enable the diagnosis of iNPH with certainty and yet early treatment with ventriculoperitoneal (VP) shunting can reverse symptoms and stop disease progression. Vascular circulation abnormalities in iNPH may be reflected by changes in subfoveal and peripapillary choroidal thickness (PPChT). This study uses spectral domain-optical coherence tomography (SD-OCT)-based measures of retinal and choroidal thickness to test this hypothesis and to assess ophthalmological non-invasive markers for iNPH.

**Methods:**

Twelve patients who displayed neurological and neuroradiological characteristics of iNPH were subject to a full ophthalmological examination including enhanced depth imaging (EDI) SD-OCT. Of the 12 included iNPH patients, 6 had undergone VP shunting with beneficial outcome. Parameters studied with EDI SD-OCT were macular retinal thickness (MT), subfoveal choroidal thickness (SFChT), retinal nerve fiber layer thickness (RNFL), and PPChT. Results were compared with 13 healthy, age-matched controls.

**Results:**

Macular thickness and RNFL and MT values of iNPH patients did not reflect atrophy. Non-shunted iNPH patients showed significantly lowered median PPChT and SFChT values compared to healthy controls. Shunted iNPH patients displayed a significantly higher median PPChT and SFChT compared to non-shunted iNPH patients. SFChT and PPChT values in shunted patients were not significantly different to values in healthy controls.

**Conclusion:**

Although limited by small sample size, SD-OCT measures in this study reveal significant changes of choroidal thickness and support the hypothesis of choroidal susceptibility to hemodynamic alterations in iNPH. Non-shunted iNPH patients in this study show choroidal thinning in combination with normal RNFL and MT values. In addition to neurological and neuroradiological exams, this pattern may aid in the challenging diagnosis of iNPH.

## Introduction

Normal pressure hydrocephalus (NPH), also called Hakim–Adams Syndrome, was first described in 1965 as a disease of cerebrospinal fluid (CSF) dynamics (1). Clinically, it is characterized by a slow, yet, progressive onset of gait disturbances, dementia, and urinary incontinence combined with indistinctive mean CSF-pressure levels and indicative neuroradiological features (2). NPH may be idiopathic or secondary to other causes like subarachnoid hemorrhage, meningitis, or previous neurosurgery. The clinical symptoms of NPH overlap with many other disorders making it a rare, yet, possibly under-diagnosed disease (3–5). Early treatment with repeated lumbar puncture (LP) or ventriculoperitoneal (VP) shunting is able to reverse symptoms and stop progression in NPH patients while other diseases that show symptoms of gait disturbances and dementia are much more difficult to treat (1, 6). Therefore, markers to specifically distinguish NPH are of high clinical relevance.

About 80% of patients with positive neurological and positive neuroradiological signs of a disproportionately enlarged subarachnoid space hydrocephalus (DESH) are described to show a response to VP shunting (7). Yet, DESH features can also present in patients who display no clinical symptoms of NPH and, to date, non-invasive clinical and radiological markers are still insufficient to enable the diagnosis of NPH with certainty (2, 8, 9). The clinical response to invasive spinal tap testing and characteristically heightened corticospinal fluid pressure wave amplitudes in the lumbar infusion test correlate well with favorable response to CSF shunting and both tests remain the gold standard for diagnosing NPH (10, 11). The final diagnosis of NPH is confirmed by amelioration of symptoms in response to CSF shunting surgery.

Several studies point to an ophthalmological involvement in NPH: a recent retrospective study found that the prevalence of glaucoma was more than triple in NPH patients (12). The translaminar pressure gradient (TLG) defined as the difference between intraocular pressure and intracranial CSF pressure (ICP) across the lamina cribrosa (LC) within the optic nerve head complex is implicated in the pathogenesis of glaucomatous changes of the optic nerve and retinal ganglion cells and, it is suggested that oscillations of high ICP wave amplitudes seen in NPH may cause optic nerve damage (13, 14). Furthermore, ICP wave amplitudes are known to have characteristic vascular effects, among them, an altered cerebral vascular compliance (15). Spectral domain-optical coherence tomography (SD-OCT) is ideally suited to non-invasively investigate retinal and vascular choroidal changes with high accuracy (16–19).

In current practice, due to its invasiveness, spinal tap and/or lumbar infusion testing is often not performed when other diagnoses seem clinically likely. The goal of this study is, therefore, to investigate the hypothesis of choroidal alterations in NPH patients using SD-OCT-based measures of choroidal thickness and to evaluate measures of both retinal and choroidal thickness as additional non-invasive markers for idiopathic normal pressure hydrocephalus (iNPH).

## Materials and Methods

The conducted study was focused on iNPH patients and carried out as an observational case-control study in an institutional setting. A total of 22 eyes of 12 consecutive patients diagnosed with iNPH between January 2009 and September 2015 were studied.

The control group consisted of 26 eyes of 13 sex and age-matched volunteers. Volunteers had no history or evidence of systemic or ocular diseases except for minor refractive errors.

### Patient Selection Criteria

Cases with high probability of iNPH or confirmed iNPH were to be assessed in this study. The electronic medical records of São João University Hospital were reviewed using the International Classification of Diseases (ICD-9) codes for “communicating hydrocephalus” and/or “occult hydrocephalus” to identify potential cases of iNPH between January 2009 and September 2015. The electronic search detected 127 cases.

A neuroradiologist (TS) blinded to the patient’s medical records independently evaluated the magnetic resonance (MR) and/or computed tomography examinations of all 127 potential cases of iNPH based on the ICD-9 search. In VP-shunted patients, imaging previous to the shunting procedure was reviewed.

**Figure 1:**
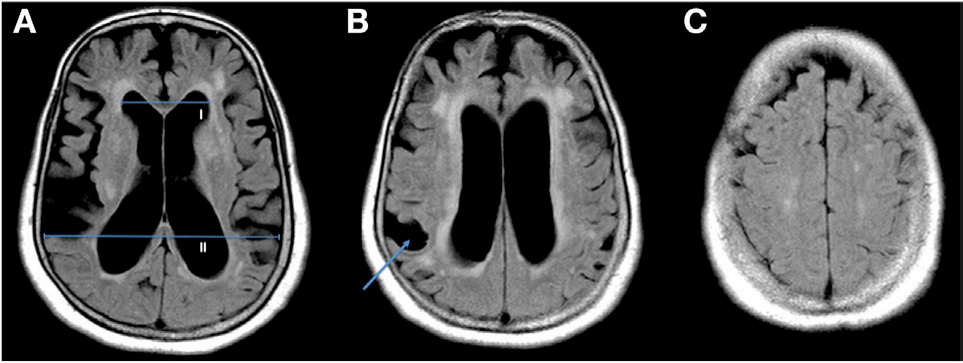
**Shown are three axial slices of a cerebral MRI from a single non-shunted idiopathic normal pressure hydrocephalus patient with ventriculomegaly**. The Evans index (EI) is calculated by dividing the maximal diameter of the frontal horns of the lateral ventricles [**(A)**, I] by the maximal diameter of the skull [**(A)**, II], values above 0.3 are considered hydrocephalic (20). Disproportionately enlarged subarachnoid space hydrocephalus features additionally consist of a focal enlargement of the cerebral sulci [**(B)**, arrow] and tight high-convexity **(C)**.

Neuroradiologically included patients were required to present at least two of the three radiographic features suggestive of DESH (Figure 1). In addition to a communicating hydrocephalus with ventricular enlargement defined by an Evans index greater than 0.3 (20), neuroradiological DESH features consist of a tight high-convexity (TC), as well as a focal enlargement of the cerebral sulci (FES) and correlate with clinical response to CSF shunting. Clinical history and records of all 127 patients were independently reviewed: all patients had to have been examined by an in-house neurologist and were required to fulfill the neurological characteristics of “probable iNPH” defined by the international criteria (2). Thus, patients had to exhibit a slowly progressive onset of two or more neurological symptoms suggestive of iNPH—gait disturbances, cognitive impairment, and urinary incontinence—over a period of at least 3 months and had to be above 40 years of age. A carried-out tap test at the time of examination was not required as an inclusion criterion for this study. However, had a tap test previously been performed, patients with reported opening pressures above 24 cmH_2_O were excluded from the study.

A history or neuroradiological signs of secondary neuropathological processes as, e.g., subarachnoid hemorrhage, intracranial bacterial infection, mass lesion, or previous neurosurgical procedures, was actively researched in every patient and, whenever present, led to exclusion from this study. From the initial ICD-9 search, most patients were excluded due to clinical criteria that made it unable to exclude a secondary cause for hydrocephalus or due to not meeting at least two of the three radiographic features suggestive of DESH.

A history of ophthalmological disorders leading to exclusion was retinopathies due to known other causes, e.g., diabetic retinopathy, as well as the presence of any ocular pathology other than cataract. Pseudophakia was not an exclusion criterion. Additionally, due to choroid changes in high myopia (21), patients with a refractive error greater than 6 diopters were excluded. Glaucoma was not considered an exclusion criterion as an association between NPH and normal tension glaucoma (NTG) is postulated in previous studies (12) and the presence of glaucoma as well as the degree of glaucomatous damage does not influence choroid thickness parameters (22–24).

### Ophthalmological Measures and Statistics

For this study, all 12 included patients and 13 healthy controls were asked to undergo a full ophthalmic examination (Joana M. Afonso). Best-corrected visual acuity (BCVA) was evaluated with Snellen charts and converted to a logarithm of the minimum angle of resolution (logMAR) chart. A slit-lamp evaluation, IOP measurements by Goldmann and iCare^®^ tonometry as well as a macular and optic disk examination were performed to exclude other ocular pathologies.

In addition, enhanced depth imaging (EDI) SD-OCT examinations of the macula and optic disk were conducted.

All OCT examinations were carried out with the same Heidelberg Spectralis SD-OCT (Heidelberg Engineering, Heidelberg, Germany) apparatus using equal parameters. SD-OCT examination of patients and controls was performed by an experienced ophthalmologist (Joana M. Afonso) and collected in one database. Consequently, an experienced, blinded ophthalmologist (Manuel Falcão) conducted choroidal measurements of both patients and controls.

**Figure 2:**
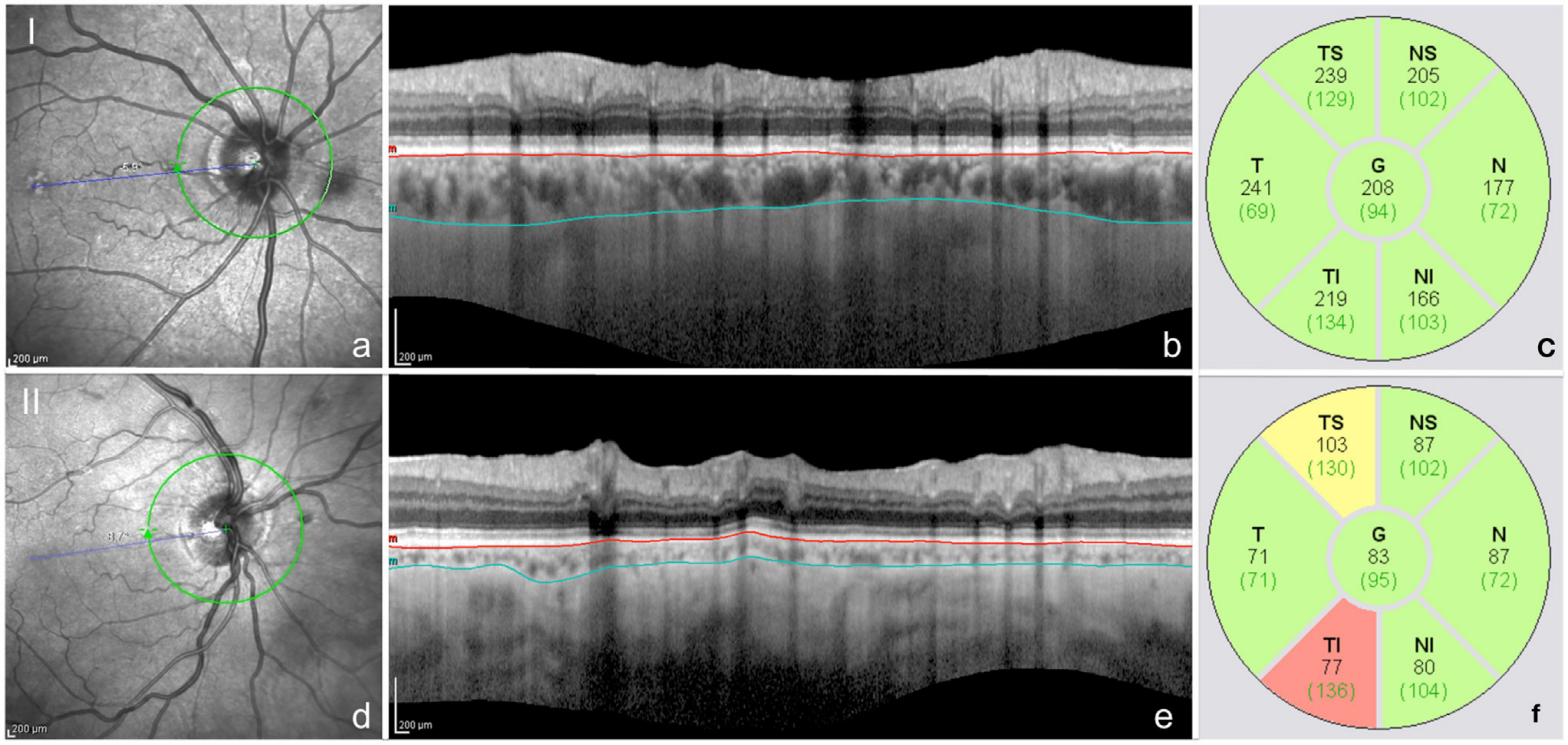
**Peripapillary choroidal thickness measurements obtained by peripapillary spectral domain-optical coherence tomography scans using the enhanced depth imaging mode (a,d)**. Images I and II correspond to the left eye of two different idiopathic normal pressure hydrocephalus patients, one after successful VP-shunting surgery (I) and another before VP-shunting surgery (II). The automatic detection of the internal limiting membrane and retinal nerve fiber layer were manually changed to delineate the external border of the retinal pigment epithelium (superior line) and the external limit of the choroid (inferior line), respectively **(b,e)**. Automatic readings of the choroidal thickness were obtained in all quadrants **(c,f)**. Choroidal thickness values are displayed in black and values of the normative database for retinal nerve fiber layer thickness evaluation of the Spectralis Heidelberg are displayed in brackets **(c,f)**.

Choroid thickness is shown to be associated with the circadian rhythm and thus thinnest at 6:00 p.m. (271.9 µm) and thickest at 3:00 a.m. (290.8 µm) (25). All patients and controls were, therefore, examined at the same daily time between 3:00 p.m. and 6:00 p.m. In each individual, a macular horizontal OCT EDI B-scan centered on the fovea, consisting of 100 averaged EDI B-scans and a macular square (20 × 20°) composed of 25 horizontal B-scans, spaced at 240 µm and averaged 20 times, was obtained. Additionally, a peripapillary scan using the glaucoma application and the preset circular retinal nerve fiber layer scan consisting of 100 average EDI B-scans was recorded. All scans were performed in the EDI mode to improve the quality of choroid imaging (26). Measurements made were macular retinal thickness (MT), subfoveal choroidal thickness (SFChT), retinal nerve fiber layer (RNFL) thickness, and peripapillary choroidal thickness (PPChT). MT was measured using the average of the central ring of 1 mm automatically determined by the Spectralis Heidelberg software. The SFChT was measured manually with the calipers provided by the same software, using a horizontal scan centered on the fovea to measure the distance from the hyperreflective line of the Bruch’s membrane to the choroid–scleral junction. Performing peripapillary scans, RNFL thickness was measured automatically using the software incorporated in the Spectralis Heidelberg. PPChT measurements were automatically performed by the aforementioned software after manually changing the automatic RNFL detection from the peripapillary area to the choroid layer limits, defined as the outer limit of the retinal pigment epithelium and the choroid–scleral junction (Figure 2). For RNFL and PPChT, the overall average value and the average values of the single quadrants and subquadrants—nasal, temporal, superior, inferior, inferior temporal, inferior nasal, superior temporal, superior nasal—were recorded. All further statistical analyses were computed with MATLAB (The MathWorks, Inc., Natick, MA, USA). Medians are given with median absolute deviation (MAD). Group statistics was primarily performed using the Kruskal–Wallis test. Since both eyes may be similarly affected by disease, the generalized estimating equation (GEE) model is a means to adjust for inter-eye correlations in paired-eye data (27, 28). All significant comparisons in group statistics underwent an additional GEE-based regression analyses using the GEEQBOX extension designed for MATLAB (29).

## Results

Twenty-two eyes of twelve consecutive patients met the inclusion criteria between January 2009 and September 2015. One eye of an included patient had to be excluded due to total retinal detachment present at the time of the study and a second eye of another patient was excluded due to a macular hole. Eight of the 12 included patients were females and 4 were males.

The studied healthy control group included a total of 13 volunteers and consisted of 9 female and 4 male volunteers (Table 1). Mean age of included patients was 75 ± 6 years and ranged between 68 and 83 years. The mean age of controls was 73 ± 7 years and did not significantly vary from patient age (*p* = 0.3295, *t*-test).

**Table 1:** **Clinical, radiological, and surgical characteristics of all 12 idiopathic normal pressure hydrocephalus patients included in the study**.

Case	Neurological triad	Disproportionately enlarged subarachnoid space hydrocephalus criteria	VP-shunt	Valve opening pressure
1	+/−/+	+/−/+	+	10
2	+/+/−	+/+/−	−	
3	+/+/−	+/+/−	−	
4	+/−/+	+/−/+	+	12
5	+/+/−	+/+/−	+	13
6	+/−/+	+/−/+	−	
7	+/−/+	+/−/+	−	
8	+/+/−	+/+/−	−	
9	+/+/+	+/+/+	+	12
10	+/−/+	+/−/+	−	
11	+/+/+	+/−/+	+	13
12	+/+/+	+/+/+	+	10

*The neurological triad is presented by order of gait, cognitive impairment, and urinary symptoms. DESH criteria are presented by order of Evans index (EI), tight high-convexity (TC), and focal enlargement of the cerebral sulci (FES). VP-shunt valve opening pressure is given in cmH_2_O at the time of the study*.

Idiopathic normal pressure hydrocephalus patients displayed a mean BCVA of 0.8 ± 0.10 on logMAR charts. Two patients were bilaterally pseudophakic with surgery having taken place more than 5 years before inclusion in this study.

At the time of evaluation, six patients presented without intracranial shunt and six patients diagnosed with iNPH had previously to this study undergone a VP-shunting procedure and placement of a programmable Codman–Hakim valve. VP shunting had been performed at least 1 year before the time of evaluation for this study and, at the time of evaluation, valve opening pressures in all six patients ranged between 10 and 13 cmH_2_O (Table 1). All six shunted patients showed a post-shunt improvement regarding gait disturbances. All five patients presenting with urinary incontinence and two of the four patients who showed cognitive dysfunction reported an additional treatment response in these respective capacities. Four of the non-shunted patients and three of the VP-shunted patients had received a documented spinal tap test between 1 and 5 years prior to the time of evaluation for this study. Normal CSF pressure and positive clinical effects were recorded in all documented cases.

Two of the included patients were diagnosed with pseudoexfoliation glaucoma (PEX) and one patient displayed NTG. At the time of the study, all three patients diagnosed with glaucoma were shunted patients.

### RNFL Thickness and Macular Retinal Thickness

Idiopathic normal pressure hydrocephalus patients and controls displayed a median RFNL thickness of 96 ± 7 µm (range, 55–122 µm) and 101 ± 5 µm (range, 89–112 µm), respectively. The difference of RFNL thickness between iNPH patients and controls was not statistically significant (*p* = 0.0974). The median MT in patients and controls measured 261 ± 29 µm (range, 198–435 µm) and 273 ± 19 µm (range, 225–338 µm), respectively. The difference of median MT between iNPH patients and controls was not statistically significant (*p* = 0.3791).

Median RFNL thickness was 97 ± 12 µm (range, 55–120 µm) in shunted and 95 ± 6 µm (range, 89–122 µm) in non-shunted patients. Median MT was 262 ± 25 µm (range, 231–407 µm) in shunted and 257 ± 31 µm (range, 198–435 µm) in non-shunted patients. The difference between shunted and non-shunted patients in both RFNL thickness (*p* = 0.9954) and MT (*p* = 0.6035) was not statistically significant.

The iNPH patient who was concomitantly diagnosed with NTG revealed mildly atrophic mean RNFL thickness values in both eyes. The two patients diagnosed with PEX glaucoma showed unilateral quadrant atrophies that did not manifest in atrophic mean RNFL values.

### Peripapillary and Subfoveal Choroidal Thickness

**Figure 3:**
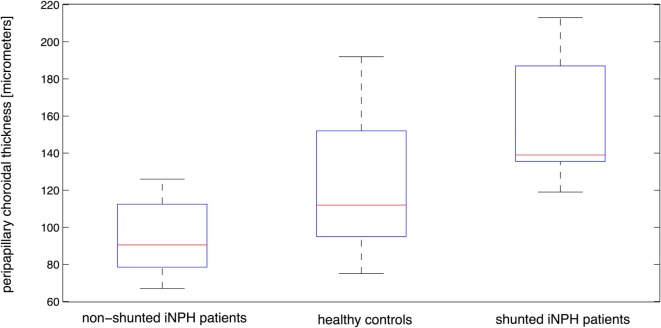
**Distribution of peripapillary choroidal thickness for shunted patients, non-shunted patients, and healthy controls with median, the 25th and 75th percentiles as well as minimum and maximum values shown for each patient group**.

Median PPChT differed strongly between iNPH patients who had undergone a VP shunting procedure and patients who had not undergone VP shunting at the time of examination. iNPH patients prior to shunting showed a median PPChT of 90 ± 15 µm (range, 67–126 µm) while patients who had undergone shunting showed a significantly (*p* = 0.0001) higher median PPChT of 139 ± 6 µm (range, 119–213 µm) (Figure 3). Controls showed a median PPChT of 112 ± 20 µm (range, 75–192 µm). Median PPChT in the non-shunted patient group was significantly lower compared to controls (*p* = 0.0272). Shunted patients displayed a higher median PPChT than controls, however, differences did not reach significance (*p* = 0.0672). Compared to healthy controls, the pattern of diminished PPChT in non-shunted iNPH patients and augmented PPChT in patients who had undergone VP-shunting surgery was consistent for quadrant and sub-quadrant measurements of PPChT. GEE-based regression analysis confirmed that PPChT differed significantly between non-shunted iNPH patients and healthy controls (*p* = 0.009). Equally, GEE-based regression analysis between non-shunted and shunted iNPH patients showed that PPChT was a significant predictor for group affiliation (*p* < 0.0001).

**Figure 4:**
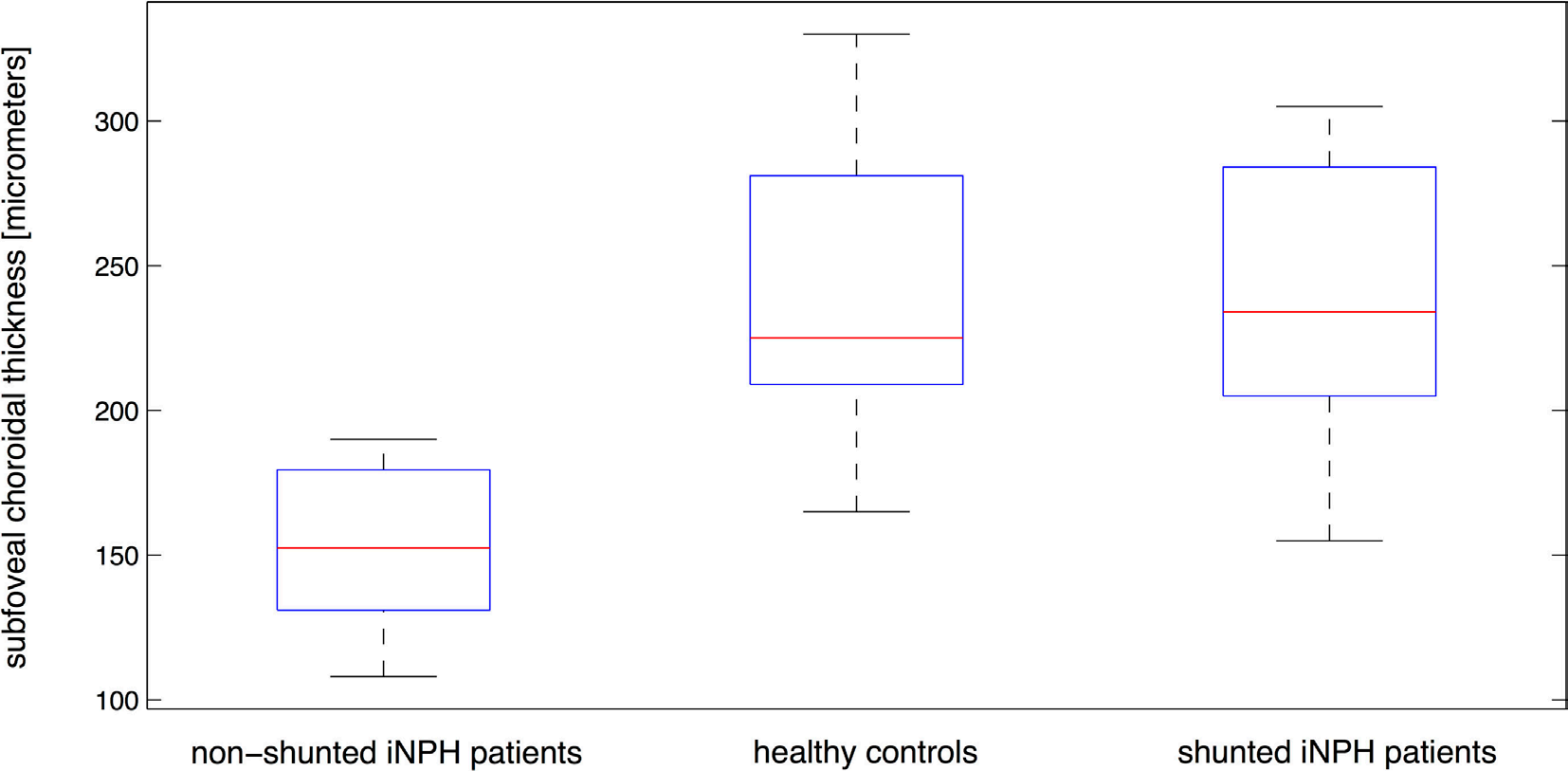
**Distribution of subfoveal choroidal thickness for shunted patients, non-shunted patients, and healthy controls with median, the 25th and 75th percentiles as well as minimum and maximum values shown for each patient group**.

Subfoveal choroidal thickness differed strongly between healthy controls and iNPH patients who had not undergone a shunting procedure, while SFChT of shunted iNPH patients was similar to healthy controls (Figure 4). Measurements of median SFChT in shunted iNPH patients were significantly higher compared with non-shunted patients (*p* = 0.0017). Median SFChT of non-shunted iNPH patients was 153 ± 23 μm (range, 108–190 µm) and significantly (*p* < 0.0001) below controls with a median SFChT of 225 ± 31 μm (range, 165–330 µm). The median SFChT of shunted iNPH patients was similar to controls and measured 234 ± 40 μm (range, 155–305 µm) (*p* = 0.9502). GEE-based regression analysis confirmed that SFChT was significantly different between non-shunted iNPH patients and healthy controls (*p* < 0.0001) as well as between non-shunted and shunted iNPH patients (*p* < 0.0001).

Medians of retinal and choroid thickness are summarized with MAD in Table 2.

**Table 2:** **Shown are median and median absolute deviation of EDI spectral domain-optical coherence tomography values of retinal nerve fiber layer thickness and macular retinal thickness (RNFL; MT) as well as peripapillary and subfoveal choroidal thickness (PPChT and SFChT) in micrometers for controls and patients with and without VP-shunt**.

	RNFL	PPChT	MT	SFChT
Shunted patients	97 ± 12	139 ± 6	262 ± 25	235 ± 40
Non-shunted patients	95 ± 6	90 ± 15	257 ± 31	153 ± 23
Controls	101 ± 5	112 ± 20	273 ± 19	225 ± 31

### Relation between Choroidal Measures and Evans Index

An Evans index (EI) above 0.3 is considered hydrocephalic, and all iNPH patients in this study showed an EI above 0.3. A mean EI of 0.41 was calculated in the whole group of studied iNPH patients as well as in the separate groups of shunted and non-shunted patients, and a GEE model-based regression analysis including all iNPH patients showed that EI did not hold significant predictive value for SFChT (*p* = 0.7472) or PPChT (*p* = 0.0658).

Comparison of median SFChT between non-shunted iNPH patients with pronounced hydrocephalic changes (defined by an EI ≥ 0.4) and non-shunted iNPH patients with less pronounced hydrocephalic changes (defined by an EI < 0.4) did not show a significant difference (*p* = 0.2337). Median SFChT in the non-shunted patient group with EI ≥ 0.4 was 134 ± 14 μm and median SFChT for non-shunted patients with EI < 0.4 was 188 ± 3 µm. In the shunted patient group, median SFChT in patients exhibiting EI ≥ 0.4 was 245 ± 18 μm while median SFChT in patients exhibiting EI < 0.4 was 223 ± 60 µm. Median SFChT showed no significant difference between shunted iNPH patients with EI above and below 0.4 (*p* = 0.9541).

Median PPChT was 87 ± 12 μm in non-shunted iNPH patients with EI ≥ 0.4 while median PPChT in non-shunted iNPH patients with EI < 0.4 was 107 ± 18 μm. PPChT did not differ significantly between non-shunted patients with EI above and below 0.4 (*p* = 0.9405). Median PPChT in shunted patients with EI ≥ 0.4 was 136 ± 9 μm while median PPChT in shunted patients exhibiting EI < 0.4 was 142 ± 9 μm. Again, PPChT showed no significant difference between the subgroups with EI above and below 0.4 in shunted iNPH patients (*p* = 0.9076).

Although not statistically significant, median PPChT and SFChT in non-shunted patients share a similar relation to EI: higher choroid thickness was seen in patients with lower EI in the studied group of patients.

## Discussion

The results of this study first revealed a significant decrease of median SFChT and PPChT in non-shunted iNPH patients compared to healthy, age-matched controls. Second, shunted iNPH patients displayed a significantly raised median SFChT and PPChT compared to non-shunted iNPH patients. And third, median SFChT and PPChT were not significantly different between shunted patients and controls (Table 2; Figures 3 and 4).

While SFChT does not seem altered by changes in venous pressure during the course of the Valsalva maneuver (30), recent studies have established a positive correlation with diastolic blood pressure as well as intracranial CSF pressure (31), and physiological diurnal variations of choroidal thickness show that the choroid is sensible to circadian and hydrostatic pressure changes (25, 32). Although the pathophysiology of iNPH is not fully understood, the characteristic elevation of CSF pulse pressure amplitude is accompanied by an indicative decrease in cortical venous compliance. Underscoring this, Bateman reported the net systolic pulse volume in the superior sagittal sinus to be distinctively lowered in non-shunted iNPH patients (15). Vascular compliance and CSF pulse pressure amplitude show a characteristic effect after VP shunting: while net systolic pulse volume is reported to increase significantly by 129% in the superior sagittal sinus (15), CSF pulse pressure amplitude is significantly decreased (33–35).

The choroid consists of choriocapillaries as well as arterious and venous vessels overlying the retina and drains into the intracranial cavernous sinus. Interestingly, choroidal vessels in this study appear to exhibit similar characteristics as the cortical venous vasculature of iNPH patients: a comparative decrease in SFChT and PPChT in non-shunted patients as well as a normal SFChT and a not significantly increased PPChT in shunted iNPH patients relative to healthy controls.

Decreased SFChT and PPChT in iNPH patients may thus reflect characteristic hemodynamic changes related to the pathophysiology of iNPH. Compared to healthy controls, the median SFChT and PPChT of non-shunted iNPH patients were reduced by 72 µm (32%) and 22 µm (20%), respectively. The choroid is thickest subfoveally (36) and although both, the subfoveal as well as the peripapillary choroid seemed thinned out in iNPH, the subfoveal area appears to display comparatively more profound alterations in the studied iNPH patients. Thinning of the choroid is often interpreted as a sign of atrophy and vessel loss (37, 38). However, shunted iNPH patients showed median SFChT and PPChT to be significantly augmented by 82 µm (54%) and 49 µm (54%), respectively, when compared to non-shunted patients in this study. A possible explanation could be that analog to augmenting cortical venous compliance (15), VP shunting may raise choroidal vessel compliance in iNPH patients and thereby lead to a distinct augmentation of choroidal thickness. In iNPH patients, it remains generally difficult to neuroradiologically assess anatomic CSF-shunting effects in clinical routine. Concordant with other studies (35, 39, 40), mean EI in shunted and non-shunted patients did not differ in the studied patient group and only complex imaging approaches are able to detect a partially reversed deformation of the brain or an augmented cortical venous compliance as a response to CSF shunting in iNPH patients (15, 41). Therefore, simple alternatives to monitor the effect of CSF shunting in iNPH patients are needed. Although the results of this study could indicate a susceptibility of PPChT and SFChT to CSF shunting in iNPH patients, longitudinal studies are needed to determine PPChT and SFChT as parameters connected to treatment response.

When VP shunting is performed, some iNPH patients show only a partial clinical response especially regarding dementia and urinary incontinence (42). This may be caused by an underlying neuronal involvement and degenerative neuronal changes in iNPH. However, regardless of reduced median PPChT and SFChT values that could imply reduced choroid nutritive functions, median RNFL and MT values in the studied non-shunted and shunted iNPH patients were not significantly different from healthy controls.

Individually, three patients in this study showed retinal atrophy mirrored in a reduced RNFL thickness in at least one unilateral quadrant. All of these cases were among the group of shunted patients and showed typical glaucomatous cup-to-disk ratios. However, possibly in part due to the limited number of patients, median RNFL values of the group of shunted patients do not reflect a significant difference compared to non-shunted patients or controls. In one of the three patients, PEX glaucoma was diagnosed prior to VP shunting. The two remaining patients with glaucomatous changes—one with PEX glaucoma and the other with NTG—were diagnosed only after VP shunting. However, it remains retrospectively indeterminable if glaucomatous changes were present before surgery. Pathophysiologically, NTG is described to be related to a heightened TLG across the LC as well as to a decreased ICP (43, 44). The LC, which is affected by ICP changes, is furthermore reported to exhibit a reduced thickness in PEX glaucoma patients (45). Thus, both elevated CSF pressure amplitudes in non-shunted iNPH patients as well as a sometimes decreased ICP in shunted iNPH patients could pathophysiologically cause distinctive glaucomatous changes of the LC and explain the heightened occurrence of glaucomatous changes in the studied group of iNPH patients. The elevated prevalence of glaucoma among iNPH patients (12) as well as the reported glaucomatous changes in visual fields of hydrocephalic children after CSF shunting (46) may support these hypotheses and further studies are needed to elucidate a possible relation.

The diagnosis of iNPH is presently based on clinical symptoms, cerebral imaging and a spinal tap, and/or lumbar infusion test (2, 8, 47). However, as non-invasive diagnostic tools alone can be inconclusive, additional non-invasive markers for iNPH are needed to facilitate the diagnosis.

Subfoveal choroidal thickness of non-shunted iNPH patients in this study never exceeded SFChT values above 190 µm. On the part of the healthy control group of this study, only two healthy volunteers displayed values below 190 μm: one showed a SFChT of 186 µm in the left eye and another of 165 µm in his right eye. However, both subjects displayed a binocular mean SFChT above 190 µm. Based on iNPH patients examined in this study, a SFChT cutoff at 190 µm would, therefore, enable a full discrimination (sensitivity and specificity of 100%) of iNPH patients from healthy subjects.

Although the measured SFChT of studied healthy volunteers was in line with age-corrected normal SFChT values published in literature (36), this cutoff estimation is limited by the small number of patients and limited age-range of patients (64–83 years) and healthy volunteers (60–82 years). Thus, the difference between SFChT values of iNPH patients in this study and age-adapted normal SFChT values based on Margolis and Spaide (36) may provide further accuracy to cutoff estimations: compared to age-adapted normal SFChT values based on Margolis and Spaide, non-shunted iNPH patients in this study display a mean SFChT reduction (*mean of both eyes*) of 38% (range 23–54%). A mean SFChT reduction of ≥23% compared to age-adapted normal SFChT values based on Margolis and Spaide, therefore, could serve as an age-adapted cutoff value with a sensitivity and specificity of 100% for iNPH patients in this study.

In spite of age adaptation, this cutoff estimation between patients and controls is limited by the small number of iNPH patients and, therefore, not generalizable. Furthermore, SFChT may be generally influenced by cognitive function (48) and possible iNPH-specific SFChT cutoffs with respect to potential differential diagnoses remain to be elucidated.

However, a combination of RNFL, SFChT, and neuroradiological imaging may prove valuable to reach higher diagnostic confidence for discrimination of iNPH from clinically or neuroradiologically similar diseases.

Disproportionately enlarged subarachnoid space hydrocephalus features identified by neuroradiological examination are used to visually differentiate iNPH from neurodegenerative types of cerebral atrophy and to rule out a hydrocephalus singularly caused by heightened mean CSF pressure (7, 49). However, disproportions of the subarachnoid space are clinically difficult to quantify and some patients with hydrocephalic ventricles show a less than clear picture. In a population-based study, Jonas et al. showed heightened CSF pressure to be physiologically accompanied by heightened SFChT (31) and, for non-shunted iNPH patients, our study showed significantly lowered SFChT values. Lowered SFChT values in possible iNPH patients may, therefore, support neuroradiological imaging by making heightened CSF-pressure as a cause for hydrocephalic ventricles seem unlikely.

Furthermore, the combination of RNFL and SFChT may aid in the differentiation of iNPH from neurodegenerative diseases, foremost Alzheimer’s disease (AD). Bayhan et al. reported a mean SFChT of 221 ± 40μm (*randomly regarding one eye per patient*) in AD patients (50). Non-shunted iNPH patients in this study displayed a mean SFChT of 153 ± 60 μm (*mean of both eyes*) with a very similar mean age of 75.8 ± 6.5 and 78 ± 6.2 years for AD and iNPH patients, in each study, respectively. Both diseases seem to feature significantly reduced SFChT values compared to healthy controls. Although SFChT reduction in iNPH patients was significantly stronger than in AD patients (*p* = 0.0008, *t*-test), a comparison of the SFChT effects alone has to be interpreted with caution as the strength of SFChT reduction is discussed to be connected to AD progression (51). However, based on the development of the OCT technique in recent years, neurodegenerative diseases like AD, multiple system atrophy, and Parkinson’s disease have all shown the similar hallmark of a significantly reduced RNFL thickness (52–58). Due to a reversal of symptoms with shunting surgery, iNPH on the other hand is not regarded a primary neurodegenerative disease and the data from this study suggests no significant effect of iNPH on RNFL thickness, especially in non-shunted patients (Table 2). A pattern of normal RNFL and MT thickness in combination with reduced SFChT and neuroradiological DESH-features may, therefore, be characteristic to iNPH patients. Detailed OCT-based segmentation of the distinct retinal layers could enable a more differentiated quantitative assessment of neuronal and axonal degeneration in future studies and confirm retinal integrity on the level of neuronal and axonal layers (58, 59).

A major limitation of this pilot study is the small sample size. Furthermore is the estimation of general specificity and sensitivity of EDI-SD-OCT features for iNPH closely linked to SFChT and RNFL cutoff values in comparison to clinically similar neurodegenerative diseases. Therefore, not only larger scale but also comparative studies are needed to confirm the outlined patterns, identify cutoff values with respect to possible differential diagnoses, and finally prove clinical utility of an ophthalmological approach to iNPH.

## Conclusion

Significantly lowered or normal choroidal thicknesses in non-shunted and shunted iNPH patients, respectively, support the hypothesis of choroidal susceptibility to hemodynamic changes in iNPH.

Although larger scale comparative studies are needed to confirm diagnostic utility of the outlined pattern, the combination of DESH features, normal RNFL, and reduced SFChT may aid to non-invasively differentiate iNPH from primary neurodegenerative diseases.

## Ethics Statement

This study was carried out in accordance with the recommendations of the ethical commission at São João University for clinical investigation with written informed consent from all subjects. All subjects gave written informed consent in accordance with the Declaration of Helsinki. The protocol was approved by the ethical commission at São João University.

## Author Contributions

JA and TS: substantial contributions to the conception, design, acquisition, analysis and interpretation of data for the work, and drafting the manuscript. MF: substantial contributions to the acquisition and interpretation of data for the work and revising the manuscript critically for important intellectual content. FS, FF-R, and SS: substantial contributions to the interpretation and analysis of data for the work and revising the manuscript critically for important intellectual content.

## Conflict of Interest Statement

The authors declare that the research was conducted in the absence of any commercial or financial relationships that could be construed as a potential conflict of interest.
